# Implementation of a peer-led school based smoking prevention programme: a mixed methods process evaluation

**DOI:** 10.1186/s12889-019-7112-7

**Published:** 2019-06-13

**Authors:** Fiona Dobbie, Richard Purves, Jennifer McKell, Nadine Dougall, Rona Campbell, James White, Amanda Amos, Laurence Moore, Linda Bauld

**Affiliations:** 1 Usher Institute, College of Medicine and Veterinary Medicine, University of Edinburgh, Edinburgh, UK; 2 UK Centre for Tobacco and Alcohol Studies, Nottingham, UK; 3 Faculty of Health Sciences and Sport, University of Stirling, Stirling, UK; 4 School of Health and Social Care, Edinburgh Napier University, Edinburgh, UK; 5 Bristol Medical School, Population Health Sciences, University of Bristol, Bristol, UK; 6 DECIPHer, Centre for Trials Research, Cardiff University, Cardiff, UK; 7 MRC/CSO Social and Public Health Sciences Unit, University of Glasgow, Glasgow, UK

**Keywords:** Tobacco, Tobacco prevention, Adolescents, Peer support, Schools, Social networks

## Abstract

**Background:**

Smoking prevention programmes that reach adolescents before they experiment with tobacco may reduce the prevalence of tobacco use. ASSIST is a school-based, peer-led smoking prevention programme that encourages the diffusion of non-smoking norms among secondary school students (aged 12–13), and was shown in a randomised control trial (conducted 2001–2004) to reduce the prevalence of weekly smoking. This paper presents findings from a process evaluation of the implementation of ASSIST in Scotland in 2014–2017. It examines acceptability and fidelity of implementation and explores the context of message diffusion between peers.

**Methods:**

Mixed method implementation study with students (*n* = 61), school staff (*n* = 41), trainers (*n* = 31) and policy and commissioning leads (*n* = 17), structured observations (*n* = 42) and student surveys (*n* = 2130).

**Results:**

ASSIST was delivered with a high degree of fidelity to the licensed manual with all elements of the programme implemented. Student survey findings indicated that the frequency of conversations about smoking increased over the ASSIST delivery period (18% at baseline, 26% at follow-up), but student recollection of conversations about smoking with peer supporters was low (9%). The delivery context of ASSIST was important when considering perceptions of message diffusion. In the study schools, survey findings showed that 0.9% (*n* = 19) of participants were regular smokers (at least once a week), with nine out of ten (89.9%, *n* = 1880) saying they had never smoked. This very low prevalence may have affected when and with whom conversations took place. Study participants indicated that there were wider benefits of taking part in ASSIST for: peer supporters (i.e. personal and communication skills); schools (an externally delivered health promotion programme that required minimal resource from schools); and communities (via communication about the risks of smoking to wider social networks).

**Conclusions:**

ASSIST in Scotland was delivered with a high degree of fidelity to the licensed programme and was acceptable from the perspective of schools, students and trainers. Targeting ASSIST in deprived areas with higher youth smoking prevalence or in other countries where youth smoking rates are rising or higher than in Scotland may be particularly relevant for the future delivery.

## Background

Tobacco use remains one of the leading causes of preventable death in the world. Globally it is estimated that 5.1 million people die each year from smoking and another 600,000 die from second-hand smoke exposure [1, 2]. In UK adults, smoking accounts for 28% of deaths from cancer and 14% of deaths from cardiovascular disease [3, 4]. Compared to their non-smoking peers, young smokers can suffer lung function and lung growth impairment and further adverse health outcomes such as lung cancer or heart disease if they continue to smoke [5]. In addition to these health impacts, smoking has also been shown to have a negative impact on longer term income and earnings, thus affecting quality of life [6].

Since the mid-1990’s the prevalence of weekly smoking has decreased in adolescents in the UK [7]. In Scotland, the national adolescent lifestyle survey has shown a consistent decline in youth smoking. For example, in 1996 30% of respondents aged 15 smoked regularly (at least once a week), in 2015, the comparable figure was 7% [8]. This reduction has largely been attributed to tobacco control policies, particularly in developed countries such as the United Kingdom (UK).

However, youth smoking rates vary by socio-economic status (SES). In Scotland, in 2015, 10% of 15 year olds living in the most deprived areas smoked compared with 5% in the least deprived. Prevalence of weekly smoking also increases with age. The younger a person starts smoking the higher the risk that they will go on to become regular smokers [9, 10]. Youth smoking prevention, therefore, continues to be an important priority for public health policy.

### The ASSIST Programme

ASSIST (A Stop Smoking in Schools Trial) is a peer-led, school-based smoking prevention programme. All students aged 12–13 years are asked to nominate the most influential students in their year group and those receiving the most nominations (top 18%) are invited to become peer supporters. The ASSIST model is different to previous school-based, teacher led, smoking prevention programmes that systematic reviews have shown to be largely ineffective [11].

ASSIST was developed in the mid -1990s, cumulating in a large cluster randomised trial of 59 schools in South Wales and Avon, England. Results from this trial showed that ASSIST was effective at reducing the prevalence of weekly smoking in young people [12, 13]. This led to the wider roll out of ASSIST in many parts of England and Wales and pilot adaptations of the ASSIST model of peer-led intervention to healthy eating and physical activity (AHEAD) [14]. ASSIST is now the subject of pilot implementations in France, Northern Ireland and Columbia, and current research is underway using this peer-led approach to encourage physical activity in young women (PLAN A [15]), drug prevention (ASSIST + FRANK [16]), to promote sexual health (STASH) and prevent gambling related harm. In a 2013 systematic review of policies and interventions to reduce socio-economic inequalities in adolescent smoking, ASSIST was identified as having a positive equity impact by reducing smoking inequalities in school children [17].

### Peer and social network approaches to health promotion

Peer-led approaches, such as ASSIST, have much to offer in the delivery of health promotion messages. Existing evidence has identified a link between peer influence and adolescent smoking behaviour [18–22]. There are also personal development opportunities for the peer supporter themselves (i.e. increased knowledge, communication skills, confidence and self-esteem) [23, 24]. In addition, peer educators are often of a similar age meaning that they are able to communicate in a less formal manner than can be achieved in teacher-led, classroom-based programmes. Evidence also suggests young people find peer-led health promotion more acceptable and credible [23, 25, 26].

ASSIST is also an example of a social network intervention in health promotion. Social network interventions have been criticised due to the limited assessment of their application to health behaviour change interventions, which means that little is known about their health promoting properties [27, 28]. In this context ASSIST is unique. It is an applied example of a network intervention using champions, grounded in diffusion theory [29], but it has also been rigorously evaluated [12, 13].

In ASSIST, opinion leaders (‘peer supporters’) are identified via ‘peer nomination’ where the whole school year complete a questionnaire to identify influential students who are then invited to become a peer supporter [30]. Peer supporters are trained to talk about the risks of smoking and the benefits of being smoke-free during everyday conversations with their peers using language and ideas that they judge will work best with the people they are speaking to. The programme consists of seven sessions, delivered over a 14-week cycle by external trainers. Table 1 summarises the key elements of ASSIST.

Existing evidence on the effectiveness of ASSIST is now over a decade old and since then the prevalence of adolescent smoking in the UK has declined. In March 2013, the Scottish Government published a new tobacco control strategy entitled ‘Creating a tobacco free generation’. The strategy included ambitious targets to reduce adult smoking prevalence in Scotland to 5% or below by 2034, and also included a commitment to pilot the delivery of ASSIST in Scottish schools [31]. This paper presents findings from an evaluation of the implementation of ASSIST in Scotland. It examines the fidelity and acceptability of implementation to the licenced manual, and explores the importance of context when considering perceptions of message diffusion between peers. In doing so it adds to the existing evidence base on the implementation of ASSIST but also, more broadly, to the delivery of school based health promotion programmes using peer support and social networks.

## Method

The implementation study involved a mixed method process evaluation with a sample of 20 schools from three National Health Service (NHS) Boards in Scotland. In two NHS Boards any school (within the Board area) was eligible to participate in the pilot delivery of ASSIST, in the other NHS Board schools were targeted based on location and available resources. A range of schools from different geographical and rural/urban contexts were selected using non-probability sampling techniques. When the study commenced the exact number of schools set to receive ASSIST (and when) was still evolving. This meant that random sampling was not possible due to the sampling frame being incomplete.

A two tier research design was used. **Tier one** comprised of three elements. First was pre and post consultation with school leads (teaching staff or deputy head teachers) who helped set up ASSIST in their school via in-depth interview (*n* = 41 interviews). Second, all trainers who delivered ASSIST were invited to take part in either a face to face interview or a group discussion, which was also conducted at baseline and follow up (*n* = 31). Last, was a repeat cross-sectional survey of students aged between 12 and 14. All students in Year 8 or Year 9 (S1 and S2 in Scotland), from schools who enrolled in the evaluation, were eligible to participate in the baseline survey. Of the 2925 eligible students 2491 (85.2%) completed a questionnaire. At follow-up (12–14 weeks later) only those students who completed the baseline survey were eligible to take part, of whom 2130 (85.5%) took part representing 14.5% lost to follow-up. Where possible the student survey was administered by the research team via a special assembly for the whole year group.

**Tier two** identified six case study schools (two from each pilot site, selected from the 20 tier one schools) where a researcher observed the entire cycle of ASSIST delivery, examining intervention fidelity and consulting with peer supporters and other students who were not peer supporters, via 12 mini group discussions (*n* = 61).

Finally, a range of stakeholders (*n* = 17) were purposively selected and interviewed either face to face or by telephone. Strategic stakeholders were selected in consultation with the advisory group and chosen because they had some experience or understanding of the ASSIST model and/or could offer comment on its applicability beyond the pilot.

The study was approved by the University of Stirling Ethics Committee (reference: 302013/14) and written informed consent was obtained from school leads and trainers. Parents/carers were given written information about the study in advance and an opportunity for their child to be opted out of the research. Student assent was also obtained. All interviews and focus groups were audio-recorded and transcribed verbatim.

### Analysis

Analysis of qualitative interviews and focus group discussions were conducted using a structured thematic approach [32] based on systematic coding of verbatim transcripts which were organised and managed via QSR Nvivo 11. Coding frames were jointly developed, piloted and amended by members of the research team. Student survey data were entered into MSExcel. The data were imported into Stata V14, assessed for data quality and consistency before undertaking descriptive analysis. Case study observations were recorded using a structured observation form which enabled recording of data on all four stages of ASSIST delivery (peer nomination, peer recruitment meeting, training days and follow-up sessions). The observation template was piloted and refined by two researchers before being used in the main study. Data from the completed observation forms were entered into an MSWord template which enabled assessment of key measures of fidelity and contributed to the thematic analysis. Findings were then triangulated across the different research strands and are presented thematically.

## Results

### Fidelity and acceptability

Fidelity measures recorded during observation of the six case study schools indicated that the programme was delivered with a high degree of fidelity to the licensed ASSIST manual. For example, all elements of the programme were delivered over a 12–14 week period and the required 18% of students in the year group to be nominated as peer supporters was met in all six case study schools. Peer supporters were particularly supportive of the peer to peer delivery approach commenting that they were more likely to listen to their peers than a teacher.

*“Yeah, because when a teacher says something to you, you don’t really pay attention to it, like, when they say something an’ you’re like that, ‘Alright then,’ but then when your mate says something, like, you pay more attention, like, you’re, ‘Oh, right,’ an’ you actually have listened to him whereas a teacher, you just sort o’ blank them an’.”*

(Peer Supporter, School 5)

The style of the training was acceptable to students who described it as ‘fun’ and ‘interactive’ which encouraged them to mix with students who they previously may not have interacted with, giving them an opportunity to make new friends.

*“Yeah, like, when you have to, like, go up in a line, like, you could work with different people... cos, like, normally all the girls would go together an’ all the boys would go together. Like, we wouldn’t mix together……it was good because you made new friends.”*

(Peer Supporter, School 1)

However, there were influencing factors perceived to have an impact on fidelity and acceptability, such as school contribution to delivery of ASSIST; the role of school chaperones and peer nomination.

Schools were not required to cover any of the intervention delivery costs but were expected to be a contact point for the trainers and help organise delivery of the programme (e.g. organise chaperones for the two-day training, secure class time and rooms for recruitment and follow-up meetings). Generally, schools found this to be an acceptable requirement, with organising chaperones (i.e. school staff) for the two-day training viewed as the most burdensome requirement. Out of the six case study schools, staff were consistently present in four schools. In one of the remaining two schools, staff were present at the follow-up sessions only and in the other, one member of the staff was present at recruitment and training sessions, but not at the in school follow up sessions. This made the trainers’ role harder, especially in relation to behaviour management. For example, during one observation of peer supporter training, the behaviour of peer supporters was very disruptive, yet school chaperones did not offer any assistance. Instead, school chaperones reported the poor student behaviour to the Head Teacher and some parents were contacted. Trainers would have preferred the school chaperones to have got involved at the time, rather than retrospectively. Similar findings were also reported in the original process evaluation of ASSIST (Holliday et al., 2009).

Challenges were also noted around peer nomination. Ideally peer nomination should be conducted under ‘exam conditions’ (i.e. students sit at individual desks in rows and do not talk to one another) either on a class by class basis or by whole year group. However, in practice exam conditions were rarely accommodated as this was overly burdensome for schools to arrange.

*“Very rarely have we managed to have them at individual desks and that’s just practicalities within schools, they don’t have the time and the feasibility to set-up the assembly halls like that. There’s been one or two but that’s quite, like near exam time because it’s been like that anyway.”* (Trainer, Site 1)

In general, schools found the peer nomination approach acceptable and understood that students with the most nominations should be invited to take part regardless of their academic ability or behaviour in school. However, there were some schools who had concerns about the suitability of some students to take part (mainly about their behaviour reflecting badly on the school) and wanted to maintain a degree of control over who took part.

*“Then I saw the list an’ I just thought, ‘Oh God, no, not on your life.’….There was about three – two or three – we had to withdraw cos there was absolutely no way on earth.”*

(School lead, School 9, baseline interview)

Student opinion from mini-group discussions regarding the acceptability of students nominated to become peer supporters was mixed. On the one hand, there was a view that those who were nominated did represent their year group because of the various friendship groups. However, there were also a belief that some students did not ‘*deserve’* to be nominated because their motivation to take part was perceived to be getting out of classes, with other more deserving students left out.

*Interviewer: Do you think the people that were picked were suitable to be picked?*

*Respondent 3 Some of them…..I think other people were just picked cos their friends picked them.*

*Respondent 1 Yeah.*

*Respondent 2 Yeah, some o’ them I don’t think deserved to go.*

*Respondent 3 But some o’ them were, like, they were up for it an’ that.*

(Students, non-peer supporters, School 4)

### Message diffusion

ASSIST encourages informal peer-to-peer diffusion of non-smoking norms by training students to have conversations with their peers. Analyses suggest that there was a degree of ambiguity regarding the extent of message diffusion, i.e. conversations about smoking.

*“I think that without a doubt, they’ve [peer supporters] learned the skills to be able to have these conversations with their peers. I think they have grown in confidence. I don’t know if they are actually doing that [having conversations]”.*

(School Lead, School 2)

This uncertainty was also found in results from the student survey. On the one hand survey responses indicated that the volume of conversations about smoking had increased over the ASSIST delivery period. At baseline 18% of students reported having conversations with friends from school about smoking in the last week. By follow-up this had increased to 26%. This suggests that smoking conversations in general between students (albeit not necessarily facilitated by peer supporters) had increased over the intervention period. However, results from the follow-up survey also found that student recollection of conversations about smoking with peer supporters was low. Nine percent (*n* = 145) of respondents answered yes, when asked if a peer supporter had spoken to them about smoking in the last few weeks.

Analysis of qualitative data suggests potential explanations for this uncertainty regarding message diffusion. First, the theory underpinning ASSIST (i.e. informal message diffusion during conversations between peers) means that conversations are not visible or obvious and therefore difficult to recall and record in a quantitative manner. This was recognised by a school lead who acknowledged that just because they were not aware of informal conversations taking place did not mean they were not happening.

*“Just because they’re not filling in the diaries, they might still be covering the conversations.”*

(School lead, School 5, follow-up)

Next are factors related to the context in which ASSIST was delivered in Scotland. Moore et al. (2015) [33] describe context as including ‘*anything external to the intervention that may act as a barrier or facilitator to its implementation, or its effects’.* Understanding the context in which an intervention is situated is, therefore, crucial to understanding how an intervention works or does not work and assessing its applicability to different settings or populations [34].

In our evaluation of ASSIST context was considered in three ways: adolescent smoking prevalence; peer supporter conversations; and mode of data collection.

#### Adolescent smoking prevalence

As noted in the introduction ASSIST was delivered in Scotland in the context of low smoking prevalence amongst adolescents. In the study schools, survey findings showed that 0.9% (*n* = 19) of participants were regular smokers (at least once a week), with nine out of ten (89.9%, *n* = 1880) saying they had never smoked. This is lower than the national average (2% of 13 year olds and 7% of 15 year olds in Scotland classed as regular smokers in 2015 [6]) and could suggest that young people may have felt the topic was not immediately relevant to them or their peers.

#### Who peer supporters were speaking to

Peer supporters also mentioned that they had engaged in conversations with other people outside of their year group such as their parents or other family members, which the survey did not collect data on. Some peer supporters reported that these conversations had contributed to friends or family cutting down or trying to stop smoking altogether. While others reported being dismissed by parents who felt that they were already aware of the dangers of smoking.

*“I’ve spoken to one of my brother’s friends, he is in fifth year and he smokes and I’ve kind of like got him to stop smoking as much. Like he used to have like one every day but now he only has like a couple every so often”.*

(Peer Supporter, School 5)

*“I done it wi ma Mum an’ Dad, an’ then they just got sick o’ me. She just told me to shut up. ‘Go to your room.’ An’ I went, ‘I’m just telling ye the facts’ an’ she was, like, ‘Well, you’re puttin’ me off.’ I went, ‘I’m meant tae.’”*

(Peer Supporter, School 1)

#### Mode of data collection

The final point concerns the context in which the data regarding message diffusion was collected which was a self-complete survey. The question that collected these data was worded as *“…in the last few weeks, has anyone who was a peer supporter talked with you about smoking?”* This required students to know who was and who was not a peer supporter. As noted by Audrey et al. [35] a key component of the ASSIST model is that students (i.e. non peer supporters) do not explicitly know about ASSIST and, therefore, may not have known who were and were not peer supporters. The authors also noted that some peer supporters chose not to reveal their peer supporter status when they had conversations with other students. Students were also asked to complete the follow-up survey at the end of the 14-week delivery cycle and may, therefore, have forgotten about conversations which took place at the start (when they were most likely to occur). These factors add to the complexity of trying to assess the extent of message diffusion, especially via a self-complete survey where recognition and recall may be compromised.

### Wider benefits of the ASSIST programme

There was a strong belief from school leads and stakeholders that ASSIST offered far more than just smoking prevention, with diffusion stretching further than the school year group and into the wider social networks of the peer supporters.

*School lead: They were speaking to folk in their clubs and their wee youth groups so I suppose you get a range of ages in there, so you did get that and as I say it was granny, grandads, mums and dads that were getting it you know, it was their family members so I dare say it was their big sisters and big brothers in there as well.*

*Interviewer: So the clubs and the youth group, was that within the school or was that out-with the school?*

*School lead: No that [ie the clubs and youth groups] was outside, outside in the community.*

(School lead, School 3, follow up)

School leads also noted that the personal and transferable skills peer supporters had developed would not only help them in school but also when they moved onto higher/further education or employment. The perceived benefits included: improved self-confidence; self-esteem; self-worth; leadership; working as a team; communication; social skills and new friendships. There was also a view that taking part in ASSIST may encourage students to sign up for other activities within the school, especially the quieter and/or less academic students who did not normally put themselves forward. Similar views were expressed by peer supporters who stated that one of the benefits of being involved in the programme was that it allowed them to make friends and grow closer to other students who had attended the two-day training course. Many also mentioned how the programme had helped to make them more confident when speaking to other people and improved their communication skills.

*“I am a lot more confident talking to people around my age group and being able to talk out of my comfort zone about something that ah didn’t know about as much before.”*

(Peer Supporter, School 3)

Stakeholders could also see the positive outcomes of ASSIST for peer supporters in terms of life skills e.g. personal and social skills.

“*When you hear about it, that they [peer supporters] get something really positive out of it which must be worthwhile. So I think that is, and I am talking about self-esteem, self-confidence, those personal qualities that people are getting out of it I think is really worthwhile.”* (Stakeholder 8)

## Discussion

The purpose of this mixed method process evaluation was to examine the implementation of ASSIST for the first time in Scotland. In particular, its primary purpose was to assess fidelity and acceptability to the licenced programme in a different country and context to the original trial.

ASSIST in Scotland was delivered with a high degree of fidelity to the licensed programme and was acceptable from the perspective of schools, students and trainers. For schools, ASSIST is one of a few evidence based programmes which requires limited school investment. Given the competing priorities and demands that schools face it is not surprising that they would support an evidence-based and non-resource intensive programme such as ASSIST. A similar result was found when schools were asked about their participation in the original process evaluation of the ASSIST programme in England and Wales [36].

BPU97112ASSIST is an informal peer led, social network intervention. To our knowledge no other informal school-based intervention like ASSIST exists. There was strong support for ASSIST which was viewed as more than a smoking prevention programme. The theory that underpins ASSIST – message diffusion using peer delivery – equips schools and students with skills and resources that extend beyond smoking prevention. For students, acting as peer supporters, ASSIST helped to build self-confidence; offered skills in communication, team working and leadership that could be transferable to a range of settings and audiences. For schools, ASSIST offered opportunities to build social cohesion amongst the school year group and a pool of students trained in peer support that could be used to promote other health related activities within the school. Taking part in ASSIST may also give schools the opportunity to strengthen existing, or form, new links with health and local authority staff, offering potential for further collaboration on other health-related issues.

This study was not intended to look at the impact of ASSIST on adolescent smoking prevalence (an implementation trial would be required to assess this [37]) but there was some uncertainty regarding the extent of message diffusion amongst peer supporters and peers in their school year. Despite an 8% increase in conversations about smoking over the intervention delivery period, student recall of conversations about smoking with a peer supporter was 9%. This is lower than the comparable figure of 23.8% when the same question was asked, at the same time period, in the process evaluation for the previous definitive RCT evaluating ASSIST [35]. Considering that ASSIST is underpinned by diffusion theory this uncertainty regarding the extent of message diffusion is not surprising; conversations are meant to be ad hoc and informal which makes them difficult to recall and quantify. Context was also an important factor when considering message diffusion. For example, ASSIST was delivered in Scotland in the context of a low adolescent smoking rate which may have affected how frequently conversations took place.

Delivery of ASSIST Scotland was not specifically targeted in deprived areas where smoking prevalence is higher than less deprived areas. Analysis of the Health Behaviours in School-aged Children (HSBC) data found that, across 35 European and North American Countries, adolescents from low affluent families were more likely to be weekly smokers than those from high affluent families [38]. This suggests that future smoking prevention policy should consider targeting delivery of ASSIST to schools in the most deprived areas. Further consideration is also merited regarding the applicability of ASSIST to low and middle incomes countries where adolescent smoking remains high [39–41].

The ASSIST Scotland evaluation suggests that message diffusion was not limited to the school year, but extended to peer supporter wider networks (e.g. family and friends). Recent research by Dobbie (2018) who mapped the social networks of 16 peer supporters found that half of conversations were with people outside of school (e.g. family members, neighbours and non-school friends) [42]. Future research and smoking prevention policy could explore the potential strengths and weaknesses of expanding the ASSIST model to peer supporter’s wider social networks, rather than just their school year. Other modes of communication to diffuse information and record conversations may be worthy of consideration, e.g. smart phones or a dedicated website. Social media platforms such as Facebook, Instagram, or Twitter may also be potential communication avenues. However, alternative approaches to record conversations have been tested in other peer supporter interventions with limited success [43] and the associated ethical implications would require careful consideration (e.g age restriction to use these platforms and any carer/parental/school concerns).

Our study has limitations. As noted by Audrey et al. [44], one of the key limitations of conducting a process evaluation is the Hawthorne Effect (i.e. the potential for research activity to influence outcomes [45]). Drawing on learning from the 2008 process evaluation, consultation with peer supporters and students was scheduled to occur towards the end of the 14 week period to avoid any influence on peer supporter activity [44]. However, our observation of an entire cycle of ASSIST introduced a risk that we may have influenced delivery and therefore our observation findings. In addition, our school sample was chosen using non-probability sampling techniques. This means that findings from the student survey are not directly comparable to the wider school population. Finally, a key component of the ASSIST model is that students are not selected by school staff but by their peers. This is deliberate to try and ensure a wide range of students with representation from students who would normally volunteer for school activities and those who would not. That said, once peer supporters were nominated it was their choice whether they chose to take on the role or not, which could result in more confident students agreeing to take. However, feedback from teachers and students was that very few students who were invited declined and that there was a good mix of students, which suggests that this was not a particular analytical concern.

## Conclusions

This study demonstrated that it is feasible and acceptable to deliver the ASSIST smoking prevention programme with a high level of fidelity beyond the context in which it was originally developed (in England and Wales). Study participants indicated that there were wider benefits of taking part in ASSIST for: peer supporters (i.e. personal and communication skills); schools (an externally delivered health promotion programme, requiring minimal resource from schools,); and communities (via communication about the risks of smoking to wider social networks). Context and the mechanism of action that involves informal conversations between adolescent peers was important when considering message diffusion. Future research areas include: exploring the utility of applying the ASSIST model to other countries with higher smoking prevalence (which is already happening in France); expanding the model to diffuse information beyond the school setting and into the wider social networks of the peer supports; and applying the model to other risk taking behaviours (e.g. gambling).

## Data Availability

The datasets generated and/or analysed during the current study are not publicly available due to confidentiality. Requests for anonymised data should be directed to the corresponding author.

## Abbreviations

ASSIST: A Stop Smoking in Schools Trial
HSBC: Health Behaviours in School-aged Children
NHS: National Health Service
SES: Socio-economic status
